# The association between residential density and physical activity among urban adults in regional China

**DOI:** 10.1186/s12889-019-7593-4

**Published:** 2019-10-07

**Authors:** Zhiyong Wang, Zhenzhen Qin, Jing He, Yuyang Ma, Qing Ye, Yaqing Xiong, Fei Xu

**Affiliations:** 1 Nanjing Municipal Center for Disease Control and Prevention, Nanjing, China; 2 Department of Otolaryngology-Head and Neck Surgery, The First Affiliated Hospital of Xiamen University, Xiamen, China; 3 School of Public Health, Nanjing Medical University, Nanjing, China; 4 Geriatric Hospital of Nanjing Medical University, 30 Rd. Luojia, Nanjing, 210024 People’s Republic of China; 5 Faculty of Geo-Information Science and Earth Observation, University of Twente, Enschede, The Netherlands; 6 International Initiative on Spatial Lifecourse Epidemiology, Enschede, The Netherlands

**Keywords:** Adults, Built environment, China, Residential density, Physical activity

## Abstract

**Background:**

Studies from Western countries reported a positive relationship between residential density (RD) and physical activity (PA) among adults. There was no such study from China, a rapidly-urbanizing country in the world. This study aimed to investigate the RD-PA association among urban adults in China.

**Methods:**

A multistage sampling approach was used to randomly select participants (aged 35–74 years old) in urban areas of Nanjing in 2017. The outcome variable was PA (dichotomized into “sufficient” or “insufficient”), while the independent variable was RD (tertiled into three sub-groups). Odds ratios (OR) and 95% confidence interval (CI) were computed to examine the RD-PA association using mixed-effects logistic regression models with adjustment for age, sex, nationality, marriage, educational attainment, employment status, body weight status, green space and neighborhood-level clustering effects.

**Results:**

Of the 1568 eligible participants, 1551 were interviewed (response rate = 98.9%), with the mean age (standard deviation) of 54.7 (11.1) years old, and 46% of men. After adjustment for potential confounders and community-level clustering effects, participants lived in communities with higher (OR = 0.31, 95% CI = 0.21, 0.47) and middle (OR = 0.70, 95% CI = 0.50, 0.99) residential density were significantly less likely to achieve sufficient physical activity relative to their counterparts lived in the lower densed communities. Similar negative RD-PA association was examined for men or women, separately. The difference in the ORs between the middle and higher RD tertiles was also statistically significant (*P* < 0.01).

**Conclusions:**

A negatively gradient RD-PA association, independent of body weight status and green space, was observed among urban adults in regional China. It has public health implications for China to help residents’ promote and maintain physical activity through planning and constructing PA−/health-friendly built environment in future.

## Background

China, as the biggest developing country in the world, has been experiencing a rapid economic growth and consequent urbanization over the past decades [1]. Urbanization usually means more and more people live in urban areas and suggests a high residential density (RD). For China, the urbanization rate dramatically increased from 17.9% in 1978 to 57.4% in 2016 [1]. Such rapid urbanization has been witnessing some health-unfriendly changes in residents’ health-related behavior patterns and lifestyles, including the declining physical activity (PA) level and the rising proportion of excess body weight among residents in China [2, 3] .

Recently, neighborhood built environment (BE) attributes have been identified as influence factors for residents’ lifestyle/behavior and the related health conditions [4, 5]. Neighborhood built environment attributes involved in health-related studies generally refer to the physical environment developed and constructed for people to live in, mainly including the following four domains: residential density, land-use mix (street connectivity, transport, sidewalks, etc.), access to destinations and aesthetics [6, 7]. Among those BE attributes, RD is a key feature that has comprehensive influence on residents’ physical activity and its related health consequences [8–10].

The majority of literature regarding RD and PA among adults is from Western countries with mixed findings. Most of the available studies showed a positive relationship between them [11–18]. However, So far, no study is from China reporting RD-PA link among adults. Considering that China is the most populous society with rapid urbanization worldwide, China may have different scenario regarding RD-PA relationship from that in Western countries. To validate the Chinese version of Physical Activity Neighborhood Environment Scale (PANES-CHN) and further to explore the association between RD and PA among adults in China, we conducted a population-based cross-sectional survey, The Built Environment and Chronic Health Conditions: Adults (BEACH-Adults), between late March and early July of 2017 in urban areas of Nanjing, China.

## Methods

### Participants and sampling approach

Nanjing, one of the mega-cities in the developed region of eastern China, was our study city, with more than 8 million registered population in 11 administration districts (6 urban and 5 rural/suburban) and a higher rate of urbanization (82.0%) relative to the average figure (57.4%) for the whole country in 2016 [19]. Under the specific context of China’s administrative system (5 strata in total: Central Government, Province/Municipality, District/County, Administrative Street/Town and Administrative Village/Residents Community), we used the residents community, the lowest and smallest stratum, as our sampling unit.

Considering our multi-stage sampling method and the two major purposes of our BEACH-Adults study, we estimated the sample size with attempt to warrant sufficient power (90%) for simultaneously validating PANES-CHN and examining associations of BE features with health conditions (overweight)/behavior (physical activity). Using parameters from our previous studies [20, 21], we estimated that approximately 1533 and 1269 subjects would be recruited for investigating RD-overweight and RD-PA relationship, respectively. Meanwhile about 1600 participants were required for validating PANES-CHN [22]. Therefore, we took 1600 as the sample size in our BEACH-Adults study. The eligible participants were local registered urban adults, without physical disability, aged 35–74 years old in Nanjing. Using a community-based multi-stage sampling approach, all these participants were randomly recruited within the 8 selected urban communities (firstly, 2 of 6 urban districts chosen; then, 4 streets from each selected district; and, next, one community/neighborhood per street selected) [22, 23]. To warrant the representativeness of participants for different age-groups (5-year interval), we chose the same number of participants as possible by age-group (totally, 8 groups), with men: women = 1:1.

Written informed consent was obtained from each participant before our survey. This study was approved by The Academic and Ethical Committee of Nanjing Municipal Center for Disease Control and Prevention. All personally identifiable information was removed prior to data analysis.

### Study variables

#### Outcome variable

PA was the outcome variable. Information on past 7-day’s physical activity time was collected using the validated Chinese version of International Physical Activity Questionnaire (IPAQ-CHN) [23, 24]. This IPAQ-CHN has been professionally translated from the original IPAQ and validated for Chinese adults [24, 25]. The approach of PA time calculation of IPAQ-CHN was the same as that used in the original IPAQ (duration× frequency per week). The sum of moderate PA plus doubled vigorous PA time was used to categorize participants into: “achieving sufficient PA (≥150 minutes/week)” or “having insufficient PA (<150 minutes/week)” in our analysis [26].

#### Explanatory variable

RD was treated as the independent variable. The number of registered residents was divided by geographic size (km^2^) to compute RD for each study community and thus all participants within a community shared the same RD. In our analysis, RD was classified as tertiles (cutoffs: 29.8 thousands and 56.5 thousands residents/km^2^). Data on registered population and geographic size for each community were obtained from local statistical department [19, 27].

### Covariates

There were some individual-level and community-level covariates accounted for in the mixed-effects logistic regression analysis, including participants’ socio-demographic and anthropometric characteristics, and public green space. As participants’ socio-demographic features, body weight status and green space have been identified to be associated with physical activity in previous studies [28–31], it is acceptable and necessary to have those specific factors considered in the analysis. Meanwhile, a neighborhood-based multi-stage sampling approach was used to select participants in our study, which might cause potential clustering-effects at neighborhood level. Therefore, potential neighborhood-level clustering-effects were also necessary to be considered using mixed-effects logistic regression models in our analysis.

The selected socio-demographic characteristics were treated as categorical variables in the analysis: gender (men or women), age (younger: 35–49, middle: 50–64 or older: 65–74), nationality (Han or others), marital status (married or others), educational attainment (≤9 years, 10-12 years or 13 + years), occupation (while collar or blue collar). All the socio-demographic information was self-reported by our participants.

Body weight status was assessed using body mass index (BMI) based on the cutoffs recommended for Chinese adults [32]. Each participant’s body weight and height were objectively measured with light clothing and without shoes inside a quite room. Beam balance scales and stadiometers were used to measure participants’ body weight and height, respectively. The readings were recorded twice and the mean value was used to calculate BMI for each participant. Body weight status was classified as: non-excess body weight (BMI<24) or excess body weight (24+).

Public green space (m^2^) refers to those green spatial areas that local residents have free access to within/around the neighborhood within 15-min walking distance. The data on green space for each study community were also obtained from local statistical department [33]. In China, local statistical authority is responsible to record and updates community level green spatial areas. Residents within a community shared the same green space in this study.

### Statistical analysis

In this study, 1568 eligible participants were recruited, while 1551 completed the survey (response rate = 98.9%). The difference in participants’ selected characteristics by gender and subgroups of residential density was compared using analysis of variance methods (continuous variables) or χ^2^ tests (categorical data). The relationship between RD and PA was examined with odds ratio and 95% confidence interval (OR and 95%CI) using mixed-effects logistic regression models. Three models were introduced: Model 1 was a univariate analysis with RD as the single predictor; Model 2 was a multivariate analysis with adjustment only for participants’ individual-level potential confounders (including age, gender, nationality, marital status, body weight status, educational attainment and employment status); Model 3 was also a multivariate analysis with adjustment for both individual-level and community-level potential confounders (green space and the clustering-effects of study community). Data were analyzed with SPSS 21.0 (IBM Corp, Armonk, NY, USA).

## Results

Table 1 displays the selected demographic characteristics of participants by gender and residential density. Among the overall participants, the mean age (Standard Deviation) was 54.7 (11.1) years old; 22.7% of participants obtained at least college level educational attainment; and 51.3% were with excess body weight (BMI = 24+). For participants within different RD sub-groups, there was no significant difference between age-groups. However, residents within higher RD tertile were more likely to have college-level educational attainment but less likely to have excess body weight.

**Table 1:** Selected characteristics of participants by gender and residential density in 2017, in Nanjing of China ^a^

	Overall	Gender		*P value*	Residential density^a^			*P value*
		Men	Women		Lower	Middle	Higher	
Number	1551	47.8 (741)	52.2 (810)		27.2 (422)	34.3 (532)	38.5 (597)	
Age (years, Mean ± SD)	54.7 ± 11.1	54.8 ± 11.4	54.7 ± 10.9	0.97	55.8 ± 10.8	54.4 ± 10.9	54.3 ± 11.5	0.08
Age group (years)								
-49 (younger)	35.9 (557)	36.8 (273)	35.1 (284)	0.10	31.0 (131)	36.7 (195)	38.7 (231)	0.07
50–64 (middle-aged)	42.0 (652)	39.4 (292)	44.4 (360)		44.1 (186)	43.6 (232)	39.2 (234)	
65+ (elder)	22.1 (342)	23.8 (176)	20.5 (166)		24.9 (105)	19.7 (105)	22.1 (132)	
Educational attainment (years)								
≤ 9	47.3 (733)	40.2 (298)	53.7 (435)	<0.01	65.5 (277)	44.4 (236)	36.9 (220)	<0.01
10–12	30.0 (466)	32.4 (240)	27.9 (226)		21.8 (92)	34.4 (183)	32.0 (191)	
≥ 13	22.7 (352)	27.4 (203)	18.4 (149)		12.6 (53)	21.2 (113)	31.2 (186)	
BMI Category (kg/m^2^)								
< 24	48.7 (755)	45.5 (337)	51.6 (418)	<0.01	42.9 (181)	47.2 (251)	54.1 (323)	<0.01
24+	51.3 (796)	54.5 (404)	48.4 (392)		57.1 (241)	52.8 (281)	45.9 (274)	
Physical activity time (mins/week)								
<150	71.5 (1109)	71.8 (532)	71.2 (577)	0.82	63.3 (267)	67.7 (360)	80.7 (482)	<0.01
≥ 150	28.5 (442)	28.2 (209)	28.8 (233)		36.7 (155)	32.3 (172)	19.3 (115)	

^a^Results presented as mean ± standard deviation for continuous data, and as percentages (number) for categorical data

^b^Residential density tertile cut-off values are 56,524 and 29,786 persons/km^2^

Table 2 presents the relationship between residential density and physical activity among participants. Relative to participants within the lower tertile of residential density, adults within the middle (OR = 0.70, 95%CI = 0.50 to 0.99) and higher tertile (OR = 0.31, 95%CI = 0.21 to 0.47) were less likely to achieve sufficient physical activity after consideration of both individual- and community-level potential confounders. Furthermore, the difference in the ORs between the middle and higher RD tertiles was also statistically significant (*P* < 0.01). The scenario of such negative gradient RD-PA association remained for either men or women after stratified analysis by gender.

**Table 2:** Odds ratio (95% CI) for being in the higher physical activity group relative to being in the lower group among urban adults in Nanjing, China

Residential density^a^	Participants in higher physical activity category (% and n/N)^b^	Mixed-effects logistic regression models					
		Model 1 ^c^		Model 2 ^d^		Model 3 ^e^	
		OR (95% CI)	*P value*	OR (95% CI)	*P value*	OR (95% CI)	*P value*
Overall							
Lower	36.7 (155/422)	1.00		1.00		1.00	
Middle	32.3 (172/532)	0.82 (0.63–1.08)	0.16	0.81 (0.62–1.07)	0.14	0.70 (0.50–0.99)	0.04
Higher	19.3 (115/597)	0.41 (0.31–0.55)	<0.01	0.41 (0.30–0.55)	<0.01	0.31 (0.21–0.47)	<0.01
Men							
Lower	33.2 (67/202)	1.00		1.00		1.00	
Middle	33.3 (83/249)	1.03 (0.57–1.89)	0.91	0.95 (0.63–1.42)	0.78	0.85 (0.52–1.39)	0.53
Higher	20.3 (59/290)	0.53 (0.29–0.98)	0.04	0.45 (0.29–0.70)	<0.01	0.36 (0.21–0.65)	<0.01
Women							
Lower	40.0 (88/220)	1.00		1.00		1.00	
Middle	31.4 (89/283)	0.69 (0.48–1.05)	0.08	0.73 (0.50–1.06)	0.10	0.61 (0.41–0.93)	0.02
Higher	18.2 (56/307)	0.34 (0.23–0.50)	<0.01	0.37 (0.24–0.56)	<0.01	0.28 (0.17–0.46)	<0.01

n: number of participants within higher physical activity category, N: total number of participants within sub-group of residential density

^a^Residential density was analyzed as a trichotomous variable (Lower, Middle or Higher tertile)

^b^physical activity was analyzed as a dichotomous variable(≥150mins/week vs.<150mins/week)

^c^Model 1 is the unadjusted model

^d^Model 2 adjusted for age, sex (overall model only), nationality, marriage, weight, educational attainment and employment status

^e^Model 3 adjusted for age, sex (overall model only), nationality, marriage, weight, educational attainment, employment status, green space and potential clustering effects at community level

## Discussion

This study examined the association between residential density and physical activity among urban adults in a rapidly-urbanizing region in China. A negative gradient association between residential density and physical activity was identified within the study participants in that the odds ratio of achieving sufficiently physical activity decreased by gradient from lower, middle to higher tertile of residential density. Our findings were inconsistent with the majority of literature from Western countries where residential density was positively associated with physical activity [11–18], but in line with our previous study conducted among urban Chinese adolescents in the same city [20].

In this study, the proportion of participants engaging in sufficient physical activity was 28.5% (442/1551), which was higher than the national average figure (22.2%) among Chinese urban adults officially reported by China Committee of Health and Family Planning in 2015 [34]. Therefore, low level of sufficient physical activity is a public health problem for Chinese people and physical activity promotion is in urgent need for China. Among those 422 participants who met the physical activity recommendations, the smallest number of persons within the categories of sufficient physical activity was 56 for women within the upper tertile of residential density. In the basis of statistical principle, such a minimal number of participants can warrant an acceptable statistical power in the data analysis. So there would be no marked adverse impact on statistical power caused by the number of participants with sufficient physical activity in our study.

Interestingly, the selected individual-level potential confounders (socio-demographic characteristics and anthropometric measures) exerted little influence on the RD-PA relationship, but the community-level factors (green space and potential neighborhood-level clustering effects) substantially mediated the RD-PA association in our study. This suggested that neighborhood-level factors might exert more important impact on local residents’ physical activity than participants’ personal socio-demographic and anthropometric characteristics. Moreover, those neighborhood-level factors markedly mediated the RD-PA relationship for women but not for men, which implied that women’ engagement in physical activity might be more easily influenced by local neighborhood-level factors relative to men. Thus, neighborhood-level covariates should be put into consideration in future studies regarding neighborhood built environment and physical activity.

The mechanisms behind RD-PA link are complicated. The most important potential explanation widely used is that an area with higher residential density, usually meaning more diverse land use and closer destinations, has more recreational facilities (stores, parks, transport stops, sport facilities, etc.) available for local residents. Thus, local residents tend to physically visit those neighborhood-around facilities or destinations by walk or bicycle [11–18], which results in that residents within higher densed neighborhoods are more likely to be physically active than their counterparts from lower densed areas. This mechanism is able to easily explain the positive association between RD and PA in Western countries, but can not explain the negative RD-PA link observed in this study.

It needs to note that the mean value of residential density within Nanjing urban areas was 9267 persons/km^2^ in 2016 [19, 27], which is unbelievably higher than that in typical urban areas of American or Australian cities where PA-related built environment attributes have been identified [35, 36]. For example, the mean residential density was 1210 persons/km^2^ in Atlanta, USA in 2010 [37]. In epidemiological studies regarding residential density and health conditions in Western communities, an area with ≥500 persons/km^2^ was usually classified as densely populated [38]. Based on this cutoff (500 persons/km^2^), RD was dichotomized as “high density” or “low density” in studies examining RD-PA relationship in Western societies, while RD was tertiled into three subgroups in our study. Furthermore, such an area with population of 500+ persons/km^2^ in Western societies might be a sparsely populated community in China. In our study, the cutoffs were 29.8 thousands and 56.5 thousands residents/km^2^ for middle and upper tertile, respectively. Even the population density within the lower tertile in our study was still much higher than 500 persons/km^2^, the cutoff for high residential density in Western countries. Thus, these may be the important factors contributing to the different scenarios of RD-PA relationship in China and Western countries.

The RD-PA relationship among adults in this study was in line with that observed within adolescents in the same urban areas in Nanjing [20]. For either adults or adolescents, RD was negatively associated with PA in urban areas of Nanjing. In those two studies, RD-PA association was examined using RD as tertiles and PA as binary variable (“sufficient” or “insufficient”). The consistent findings from those two studies suggested that RD might have similar influence on residents’ physical activity irrespective of their age in urban areas of regional China.

There was a possible explanation on such an inverse association between residential density and physical activity observed in China. One important potential inhibitor to outdoor physical activity may be roads/streets and traffic volume around neighborhoods. For those neighborhoods with high-enough population density in mega-cities like our study city, the roads/streets around neighborhoods are typically narrow and/or crowded with traffic which implying not sufficiently safe, even if not unsafe, for outdoor activities. Therefore, local residents within such highly-densed neighborhoods might not be willing to take the potential risk to do outdoor physical activities. People might choose to stay at home instead to visit parks or some places with physical activity facilities by walking a little bit long distance under such unsafe traffic-related circumstance. This might, at least in part, explain that the very population-densed urban environment may inhibit physical activity observed in our city. However the mechanisms behind RD-PA relationship are really complex and need to be further investigated with well-designed programs in future.

Regarding the strengths of this study, it is the first study reporting RD-PA relationship among urban adults from a rapidly urbanizing region in China. In this study RD was used as tertiles not dichotomous variables, which was more informative and allowed us to look at a gradient RD-PA association. The second strength is that, in addition to conventional confounders (including green public space), community-level potential clustering-effects were also considered using mixed-effects regression models. Finally, an interesting finding, a negative RD-PA association, was examined among urban adults, which was consistent with that in our previous study among Chinese urban adolescents, but not in line with those reported from Western communities, implying complex mechanisms behind the RD-PA link and suggesting further well-designed studies needed in different countries.

Several limitations also need to be noted. First, the RD-PA link did not imply any causality as it was observed from a cross-sectional study. Second, information on PA was self-reported by participants, which might cause potential recall bias, although the PA questionnaire has been validated and widely used to collect PA data in epidemiological surveys [39, 40]. Finally, although socio-demographic characteristics, public green space and community-level cluster-effects were controlled for in our analysis, some other potential confounders could not be considered due to data limitation. Therefore, considering these limitations of this study, the findings shall be prudently interpreted, and well-designed longitudinal studies are in need to demonstrate the influence of residential density on residents’ physical activity in future.

## Conclusions

Residential density is, in gradient, negatively associated with sufficient physical activity for urban adults in regional China. It has public health implications for China to help residents’ promote and maintain physical activity through planning and constructing PA−/health-friendly built environment in future.

## Data Availability

The related data and material will be available upon request to either of the two corresponding authors.

## Abbreviations

BE: Built environment
BEACH-Adults: The Built Environment and Chronic Health Conditions: Adults
BMI: Body mass index
CI: Confidence interval
IPAQ-CHN: The Chinese version of International Physical Activity Questionnaire
OR: Odds ratio
PA: Physical activity
PANES-CHN: The Chinese version of Physical Activity Neighborhood Environment Scale
RD: Residential density
